# Inspecting the Solution Space of Genome-Scale Metabolic Models

**DOI:** 10.3390/metabo12010043

**Published:** 2022-01-05

**Authors:** Seyed Babak Loghmani, Nadine Veith, Sven Sahle, Frank T. Bergmann, Brett G. Olivier, Ursula Kummer

**Affiliations:** 1 Department of Modelling of Biological Processes, BioQuant/COS Heidelberg, Heidelberg University, 69120 Heidelberg, Germany; babak.loghmani@bioquant.uni-heidelberg.de (S.B.L.); nadine.veith@bioquant.uni-heidelberg.de (N.V.); sven.sahle@bioquant.uni-heidelberg.de (S.S.); frank.bergmann@bioquant.uni-heidelberg.de (F.T.B.); 2 Systems Biology Lab, AIMMS, Vrije Universiteit Amsterdam, 1081 HZ Amsterdam, The Netherlands; b.g.olivier@vu.nl

**Keywords:** solution space, perturbation, sensitivity, robustness, constraints, FBA, FVA

## Abstract

Genome-scale metabolic models are frequently used in computational biology. They offer an integrative view on the metabolic network of an organism without the need to know kinetic information in detail. However, the huge solution space which comes with the analysis of genome-scale models by using, e.g., Flux Balance Analysis (FBA) poses a problem, since it is hard to thoroughly investigate and often only an arbitrarily selected individual flux distribution is discussed as an outcome of FBA. Here, we introduce a new approach to inspect the solution space and we compare it with other approaches, namely Flux Variability Analysis (FVA) and CoPE-FBA, using several different genome-scale models of lactic acid bacteria. We examine the extent to which different types of experimental data limit the solution space and how the robustness of the system increases as a result. We find that our new approach to inspect the solution space is a good complementary method that offers additional insights into the variance of biological phenotypes and can help to prevent wrong conclusions in the analysis of FBA results.

## 1. Introduction

Computational modelling has become a standard approach to achieve a comprehensive understanding of metabolic networks. In the absence of detailed kinetic data and in order to represent metabolism as a whole, genome-scale models based on the stoichiometric wiring of the system are a suitable way to, e.g., determine permissible and optimal flux distributions [1]. For this purpose, an optimality criterion, usually biomass production has to be defined [2] and optimal flux distributions are calculated. This process which uses linear programming is called flux balance analysis (FBA) [3]. The result is usually one specific flux distribution which, however, is by no means unique [3]. The presence of ample alternative solutions, however, limits the predictability of genome-scale metabolic models. To increase the predictability, different types of constraints can be applied [4]. Among the most commonly used constraints are consumption and production rates of metabolites in the medium [4]. Furthermore, transcriptome and proteome data have recently been used to reduce the solution space of the optimization. This data can be used to reduce the topology of the system by eliminating reactions for which no gene expression for the responsible enzyme can be found [5]. In addition, the abundancies of transcripts or proteins can be used to constrain the respective flux bounds [6,7,8].

However, even with all of the above described types of constraints, the solution space of an FBA problem remains large and FBA solutions are still not unique. There are various attempts to study properties of this solution space. In general, the solution space of a constraint-based model is convex [9], consisting of all the steady-state flux distributions under a given set of constraints and an optimality criterium. The most common approach to study the solution space in more detail is named flux variability analysis (FVA) [10]. Here, the range of flux is determined for each individual reaction for which an optimal (with respect to the optimality criterion) flux distribution of the network is found. However, this does not sufficiently provide information on the feasible combinations of individual fluxes resulting in specific biological responses.

In the past, different methods have been developed to go beyond FVA when inspecting the solution space. Kelk and colleagues developed a method named CoPE-FBA, which decomposes the alternative flux distributions into three topological features, namely vertices, rays and linealities (corresponding to paths, irreversible and reversible cycles in a metabolic network, respectively) [11]. They showed that the optimal space is often determined by a few subnetworks/modules consisting of numerous reactions with multiple internal routes in each. Having analysed the solution space via this method, the entire space can be characterised by a few subnetworks/modules. Consequently, two reactions would occur in the same module if their flux value across all vertices is correlated. These reactions might appear either in the same flux route or in exclusive ones.

Other common methods rely on sampling the solution space for the investigation of its properties. Monte-Carlo sampling of the steady-state flux distribution has been used to calculate the probability distribution of the flux values as well as the correlated reactions, revealing those sets of reactions which display a highly correlated flux [12]. It has also been reported that correlated reaction/gene sets can be considered as modules to characterize regulatory networks [13]. In another attempt, using random sampling, average values and standard deviation of all the reaction rates were used to describe the changes in flux values between different conditions and compare these to gene transcription profiles [1]. Sampling the solution space has also been used to find the steady-state flux distribution consistent with experimental data under various physiological conditions in human mitochondrion [14]. One drawback of these methods (specifically Monte-Carlo sampling) that they are computationally quite expensive, although recent development has made the process more efficient and cheaper [15]. To name one, coordinated hit-and-run with rounding (CHRR) is reportedly a very efficient method and has been used to study the properties of genome-scale models of bacterial systems [15,16]. Sampling methods have also been used to study the effect of a change in biomass precursors, ATP maintenance (ATPm) and the uptake/production rates of metabolites in response to temporal fluctuations in environments, leading to the conclusion that incorporation of such constraints considerably increases the accuracy of flux distribution [17].

An inevitable consequence of having a large solution space is that many solutions denote biologically unrealistic phenotypes. For instance, the generation of internal loops in the network might result in the interconnectivity of two electron pools, or the production of energy resources such as ATP [18]. As a result, the internal flux distribution of constraint-based models is not reliable and is considered to be one of the shortcomings of this modelling approach.

In this study, we investigated the solution space of different genome-scale models particularly focusing on inconsistencies between simulation and experimental results using a new strategy. With this strategy, we fix individual fluxes randomly to different values within the permissible interval given by FVA and recalculate FBA. In this way, we get a multitude of different optimal flux distributions in a computationally relatively cheap, but not exhaustive way. We used this approach to examine the effect of different experimental constraints on the solution space, to study the capabilities of the models to fit the experimental data. While Monte-Carlo methods have majorly focused on the analysis of individual/subsets of reactions (finding probability distributions of flux values or finding correlated fluxes), our method is designed to obtain a whole-system overview by showing the degree of sensitivity of reactions, the extent of the sensitivity/variability of such reactions, and the model behaviour at phenotype decisive branching points, by using a relatively small sample size. We also compared our findings to the results obtained using the CoPE-FBA method. The comparison was aimed at finding to what extent the results of our method are mirrored by the modules generated by CoPE-FBA. For this purpose, we used models of *Enterococcus faecalis* [19] and a knock-out mutant of glutamine synthase (Δ*glnA*) of *Enterococcus faecalis* applying different constrains, e.g., metabolic and proteomic data [7] to trace their effect on the solution space. Moreover, we employed genome-scale models of *Streptococcus pyogenes* [20] and *Lactococcus lactis* [21] in order to reproduce parts of our findings, to ensure that the results are not restricted to one specific model and to show that our findings can be considered characteristic of genome-scale models and/or the numerical routines that are employed for calculating FBAs in general.

## 2. Results

To investigate the solution space, as well as the robustness of the solutions produced by FBA, a published genome-scale metabolic model of *E. faecalis* (a wildtype [19] and a *ΔglnA* mutant variant [19]), as well as models of *S. pyogenes* [20] and *L. lactis* [21] were used. The respective analyses were aimed at revealing the dependency of the size and properties of the solution space on different constraints and parameters. The robustness of the steady-state flux distribution as calculated by FBA was examined by extensive analysis of the solution space. The complete results can be found in Supplementary file 1.

### 2.1. Effect of Constraints on the Solution Space in the Network

Initially, we used a published genome-scale metabolic model of E. faecalis [19] in its wildtype form, as well as a variant of this model, namely a Δ*glnA* mutant, in which the flux of glutamine synthetase (R_GLNA) was set to zero. For both models, experimental data on the medium composition, as well as on uptake and release of different metabolites, namely carbohydrates, organic acids (fermentation products) and amino acids, as well as dry mass measurements were available and integrated into the models step-wise. The feasible flux interval of each reaction was determined by FVA [10]. Thus, when analysing the E. faecalis wildtype and mutant models, we first counted how many reactions—with and without the given constraints—have a variable flux according to FVA (Table 1). As can be seen, for these two models (wildtype and the mutant), starting with an entirely non-constrained model, the addition of constraints at each step (constraining with the medium composition, the uptake and production rates of metabolites and finally with proteome data)—not surprisingly—results in a reduction of the number of variable reactions.

### 2.2. Inspecting the Solution Space Using Random Perturbations

Knowing the number of variable reactions and the extent of this variability is certainly important information. However, it does not easily allow insight into how variable biological phenotypes in the solution space are. To gain a comprehensive picture, we wanted to investigate how robust the steady-state flux distribution and especially the resulting conclusions are with respect to different alternate optimal flux distributions. Thus, we developed a new approach based on the FVA results: Here, fluxes which according to default parameters in FVA display a variability of flux beyond a change of 10^−6^ are considered to be “variable”. All other reaction fluxes are seen as “stable”. It is also interesting to note that the majority of reactions in all models, except for wt + med + met + pro, had an interval size larger than 10^−3^. For each reaction showing a flux interval determined by FVA, the respective flux value was fixed to 10 different randomly selected values within the given FVA interval. We will refer to this as a “perturbation”. Then, for each of the 10 randomly fixed flux values, FVA and FBA were recalculated and the effect on the flux distribution in terms of taking an alternative optimal distribution was investigated. In addition, we were interested in the extent to which reactions show a variable flux and therefore, we will refer to reactions that change their flux value in response to a perturbation by at least 5 percent as “sensitive”, and reactions that change their flux by less than that and therefore always carry approximately the same flux value irrespective of any perturbation as “robust”. It has to be pointed out that the value of ±5% resulted in an acceptable distinction between different characteristics among models within our set-up, but this can be adjusted to a higher/lower value if necessary. Please note that the concept of “stable” and “robust” are not the same, as “stable” refers to reactions for which FVA denotes no flux variability, while “robust” refers to variable reactions with a very small flux interval. We found the width of the permissible flux to be an important indicator of the size of the solution space.

**Figure 1:**
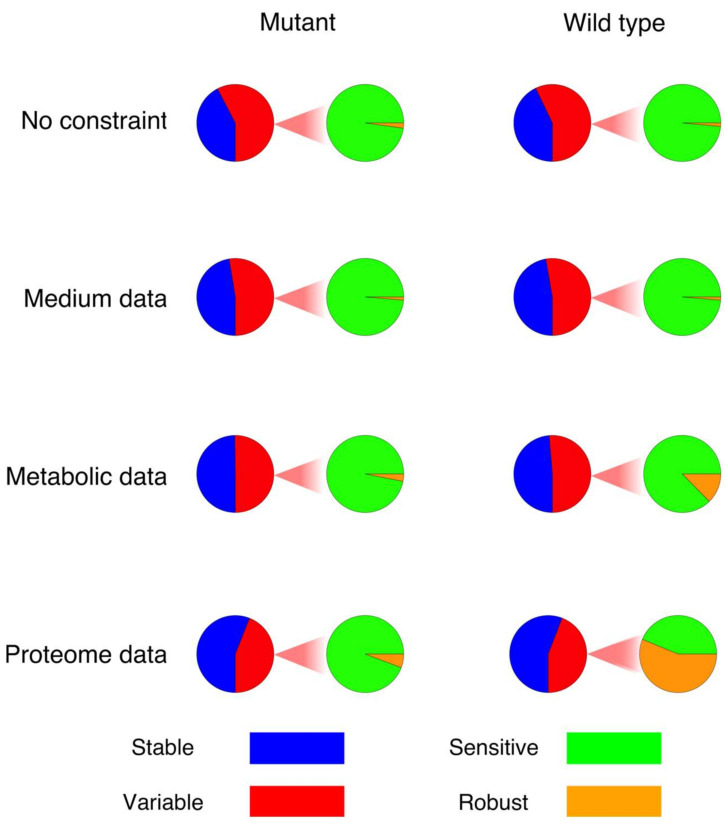
The fraction of variable reactions in each model, separated into sensitive and robust reactions. The term “robust” in this figure refers only to the fraction of variable reactions with a variability interval of less than ±5%.

The above strategy was applied to the models of *E. faecalis*, with and without the given experimental constraints. Accordingly, we first counted how many reactions in the models (wildtype and mutant) were sensitive (exhibited flux changes of more than 5 percent) after a perturbation in comparison to the original flux distribution (Figure 1). As can be seen in Figure 1, the addition of constraints results in a reduction in the number of sensitive reactions, which in turn raises the number of robust reactions. Moreover, the integration of constraints showed a quite different effect on the number of “sensitive” reactions when comparing the wildtype and mutant models (Figure 1). Interestingly, although there are more variable fluxes in the model of the wildtype according to FVA, more reactions actually changed their flux values significantly in response to perturbations (sensitive reactions) in the model of the mutant, especially after the constraints have been applied. Moreover, following the integration of constraints and consequently limiting the solution space, the number of sensitive reactions that changed their directionality in response to perturbation decreased dramatically. In the model of the mutant, the percentage of reactions showing a reversed flux dropped from 33% (mt + nc) to 13%, 7% and 3% (mt + med, mt + med + met and mt + med + met + pro, respectively). In the case of the wildtype, a similar trend was observed, with the percentage decreasing from 35% (wt + nc) to 14%, 4% and 4% (wt + med, wt + med + met and wt + med + met + pro, respectively).

In order to grasp the essence of sensitivity in constraint-based models, a more elaborate analysis of the sensitivity seems to be necessary. One of the interesting aspects of our findings was that the overall sensitivity in the network, namely the number of sensitive reactions seems to have a direct correlation with the size of the solution space. Besides that, the number of variable reactions with a variability of larger than 10^−3^ showed a similar correlation.

Here, we should also point out that it is difficult to differentiate between FBA properties in general and the employed numerical routines. In order to make sure that our results are not purely mirroring numerical behaviour of the used optimisation algorithms, we repeated the above calculations using the solvers CPLEX and GLPK as implemented in the COBRA toolbox (when flux distribution was obtained by FBA function). For FVA, we also used PySCeS-CBMPy [22] (obtaining flux distribution by FVA function, similar to the original procedure with COBRA). The result showed that despite some small differences, the trend of the decreasing number of sensitive reactions following the integration of constraints was captured by all the above-mentioned software and solvers.

Figure 1 shows the fraction of variable reactions that are sensitive or robust in each model, respectively. The integration of constraints results in an increase in the robust fraction, pointing on a significantly decreasing sensitivity interval. While the extent of this effect is very different between the wildtype and the mutant, the overall pattern seems to be conserved.

However, the extent to which sensitive reactions change their fluxes as well as the frequency of the changes seem to vary independently of the trend in which the number of sensitive reactions changes. When analysing the frequency, which is the number of times a reaction significantly changed its flux after perturbations, it was revealed that the sensitive reactions in the model of the mutant constrained with the medium composition data responded to perturbations less frequently, compared to the models with metabolic and proteome data, although the number of sensitive reactions in those models is lower (Figure 1). However, these findings show poor agreement among the results obtained by different software and the results seem to be non-conclusive and significantly affected by numerical libraries and software implementations.

**Figure 2:**
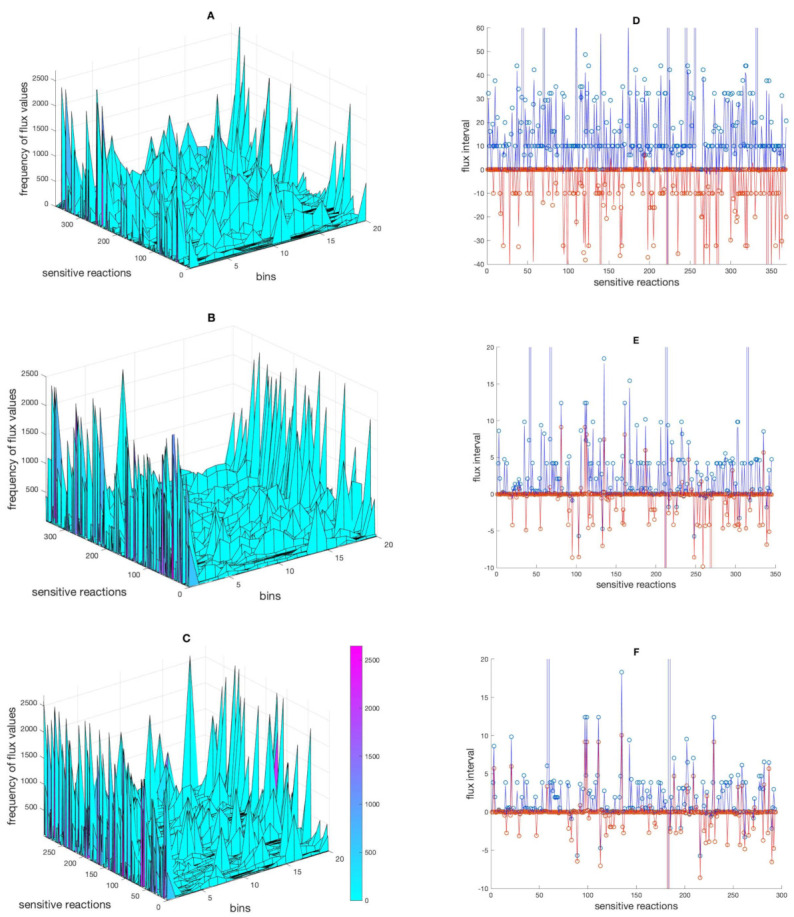
The distribution of alternative flux values across the flux intervals. Panel (**A**–**C**) show the frequency of flux values of sensitive reactions divided into 20 bins in the mt + med, mt + med + met and mt + med + met + pro models, respectively. Panel (**D**–**F**) show the intervasl in the respective models in which sensitive reactions responded to perturbations (red and blue lines, indicating lowest and highest flux values, respectively), and the interval given by FVA, indicated by red dots (lower bounds) and blue dots (upper bounds). For the sake of clarity, a few extreme points in panel (**D**–**F**) are excluded.

When performing the perturbation, the new flux value for one reaction can be very different from the original flux value in the reference flux distribution profile. To find out about the distribution of the alternative flux values taken by the sensitive reactions across the flux intervals following the perturbations, the flux ranges of sensitive reactions (in the mt + med, mt + med + met and mt + med + met + pro models) as determined by FVA were divided into 20 bins and the frequency of fluxes occurring in each bin was calculated (Figure 2A–C). Moreover, the interval in which each sensitive reaction responded to perturbations was determined and compared to intervals given by FVA (Figure 2D–F). The result showed that the responses of sensitive reactions (to perturbations) generally happen in larger intervals in the model with integrated medium composition data compared to the two models with more constraints. This is no surprise. However, it is interesting to see that the distribution of the flux values showed that with increasing constraints more and more flux values were taken that sit at or are close to the boundaries of the permissible intervals. Furthermore, the comparison of the FVA interval sizes (when larger than ±5% of the original value) with the frequency of response (the number of times that a reaction responded to perturbations) revealed no connection between the two (Supplementary Figure S1). Thus, a large permissible interval as calculated by FVA does not imply that the corresponding flux is often changing its value as a result of perturbation. Finally, the results revealed that although the frequency of response cannot be conclusively discussed (due to the significant impact of software and libraries on the exact quantity); the interval in which sensitive reactions change their flux decreased overall. This suggests that the latter, the flux interval, is a more indicative index of the size of the solution space.

The result suggests that although FVA is fundamental in the analysis of the solution space, it only captures one aspect of the solution space, which is the mere existence of alternative optimal flux values. However, it is also important to analyse the width of the permissible interval in a direct way, e.g., by the definition of sensitive reactions, which seems to be one crucial aspect when analysing the solution space.

### 2.3. Investigating Biological Phenotypes in FBA Results

In order to study biologically relevant phenotypes and their appearance in the solution space of FBA, three different metabolic branching points were analysed using the perturbation approach. Branching points are interesting in this respect, since they represent points where alternative biological phenotypes can differ significantly. Looking only at FVA results it is possible to analyse feasible flux ranges for single reactions, but impossible to extract permissible ratios between several fluxes.

**Figure 3:**
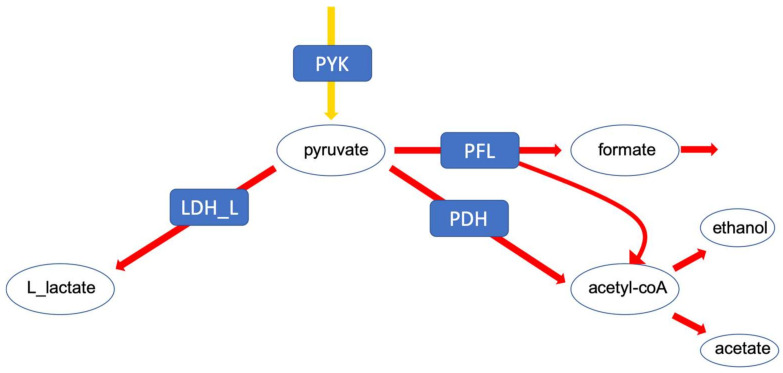
The pyruvate branching point. The distribution of flux at this point determines the fermentation profile of the cell.

First, we investigated the branching of fluxes at the level of pyruvate. In lactic acid bacteria, pyruvate can be converted to either lactate which corresponds to homolactic fermentation or to mixed acids, comprising ethanol, formate and acetate, referred to as mixed acid fermentation. This particular branching point is highly relevant for biotechnological applications (Figure 3).

**Figure 4:**
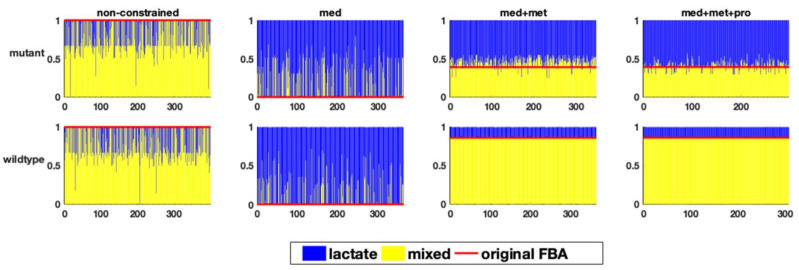
The relative flux distribution in the branching point of carbohydrate fermentation (y-axis) in response to one perturbation in each of the variable reactions (x-axis) in the two studied genome-scale models of *E. faecalis*, resulting in homolactic or mixed acid fermentation in the two genome-scale models of *E. faecalis*.

The branching point also plays an important role in determining the state of energy production in anaerobic organisms in general. Figure 4 shows, for the different models and combinations of constraints, which fraction of the flux goes towards the homolactic or the mixed acid fermentation pathway in the different sampled FBA runs.

As can be seen, the step-wise addition of constraints results in narrowing down the range of flux distributions. The non-constrained model (both for the wildtype and the mutant) predicts a highly variable flux distribution, ranging from absolutely homolactic to lactate-free fermentation. This finding is not surprising, since both types of fermentation can exist in lactic acid bacteria. Defining the medium helped to rule out a lactate-free fermentation from the solution space for both models. However, fully homolactic fermentation is still very frequently predicted, whereas a mix of homolactic and mixed-acid fermentation is observed experimentally. This is noteworthy, since FBA is frequently performed based on only data of the medium composition, which in this case, is clearly not sufficient for a reasonable model prediction. Only the integration of metabolic flux data, e.g., in form of uptake and production rates, as expected, fixes the amount of fermentation products so that the flux distribution profile can more accurately reflect the experimental data.

However, in the model of the mutant, even the integration of the metabolic flux data still allows qualitative differences in the flux distributions (Figure 4). In this case, in the original FBA of the mutant model, slightly more than 50% of the carbon flux goes towards lactate production, consistent with the experimental observations. However, a highly variable fermentation pattern was observed in the solution space of the FBA results using the perturbation approach, ranging from almost homolactic fermentation to mixed acid fermentation with less than 50 percent lactate formation. The integration of proteome data lessens this variability, but does not completely eliminate it.

The fact that the addition of the constraints in the wildtype model resulted in the elimination of non-physiological behaviour suggested that the solution space of the constrained wildtype model is more highly limited than the one of the mutant. This is consistent with the result of the above perturbation/sensitivity analysis, revealing that less reactions in the wildtype model are sensitive to perturbations in the network (Figure 1), suggesting a smaller solution space compared to the mutant model (as discussed in more detail before). It needs to be stressed again that all these flux distributions show the same value for the biomass production rate as the optimality criterium, while also fulfilling the constraints.

**Figure 5:**
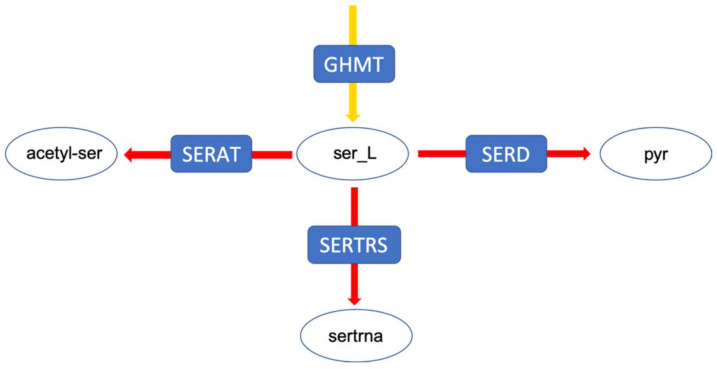
The serine branching point by which serine is distributed. Pyr: pyruvate; sertrna: L-seryl-tRNA; acetyl-ser: Acetyl-serine.

**Figure 6:**
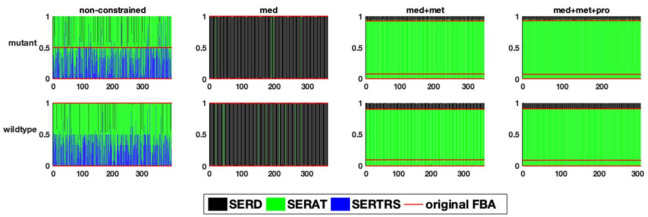
The relative flux distribution in the branching point of serine metabolism (y-axis) in response to one perturbation in each of the variable reactions (x-axis) in the two studied genome-scale models of *E. faecalis*, resulting in the production of acetyl serine, or seryl-tRNA or serine secretion.

In a second example, the flux distribution at a branching point in serine metabolism is evaluated (Figure 5). This branching point can lead both to amino acid metabolism, tRNA loading, as well as central metabolism. After integrating the medium composition, in both models, a very large fraction of the flux is predicted to be channelled towards the serine O-acetyltransferase reaction (Figure 6). However, the addition of experimental metabolic flux data results in a redirection of the flux towards the other two reactions, namely serine dehydrogenase and seryl-tRNA synthetase. Although we did not explicitly include tRNA loading in our models, it should be mentioned that there have been recent efforts to include this process into genome-scale models, which further improves the inclusion of expression data [23,24,25,26,27]. In the original FBA of the fully constrained mutant model, around 95% of the flux is channelled through serine dehydrogenase. This value can slightly vary (less than 1%) in solution space (Figure 6), however, to a much smaller degree compared to the above described flux distribution at the pyruvate branching point. It is important to note that this small variation does not exist in the wildtype model, supporting once again the suggestion of a smaller solution space in the wildtype model.

**Figure 7:**
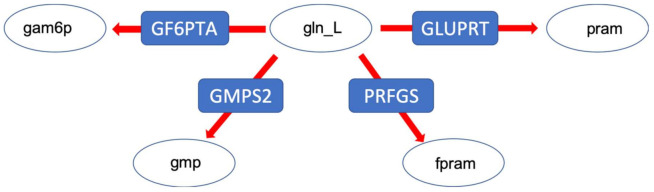
The glutamine branching point distributing amino-groups via the amino acid L-glutamine. Gln_L: glutamine, pram: 5-Phospho-beta-D-ribosylamine, fpram: 2-(Formamido)-N1-(5-phospho-D-ribosyl) acetamidine, gmp: guanosine monophosphate, gam6p: glucoseamine 6 phosphate.

**Figure 8:**
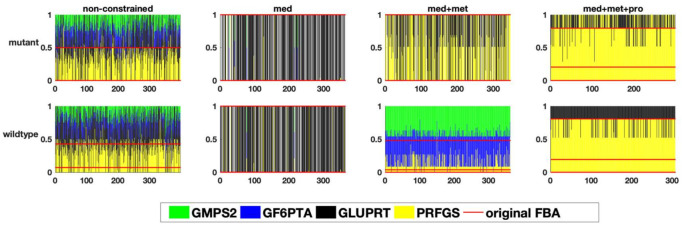
The relative flux distribution in the glutamine branching point(y-axis) in response to one perturbation in each of the variable reactions (x-axis)) in the two studied genome-scale models of *E. faecalis*, resulting in the distribution of glutamine in different pathways, namely amino acid, purine and pyrimidine metabolism.

Finally, we investigated a branching point in glutamine metabolism, where nitrogen groups are distributed throughout metabolism via the amino acid L-glutamine. Thus, the distribution of glutamine among different pathways was studied (Figure 7). Here again, the result revealed that despite a huge variability in the non-constrained models, and the ones constrained by medium composition only (Figure 8), the integration of metabolic flux data and proteome data resulted in a more determined distribution pattern and a strong re-direction of the flux towards pyrimidine and purine metabolism (by means of the PRFGS and GLUPRT reactions) in both models. In the case of the wildtype model, the integration of metabolic flux data without proteome data resulted in a very diverse flux distribution, once more signifying the importance of proteome data (Figure 8).

### 2.4. The Influence of Specific Quantitative Constraints on the Solution Space

The results of our perturbation approach suggest that the solution space of the wildtype model is indeed considerably smaller than that of the mutant. Thus, the wildtype model was more robust with respect to perturbations. The number of sensitive reactions in the wildtype model was less than half of that of the mutant model in the most constrained cases. This finding can be considered to be an informative indicator of the solution space. Moreover, analysing the flux distribution profiles at metabolic branching points revealed that there is no variation in response to perturbation among different FBA runs (in the three examples shown here) in the wildtype model, whereas in the mutant model, especially in the case of the pyruvate branching point, the variation resulted in qualitatively incorrect predictions, contradictory to experimental data.

To validate that the wildtype model better fits the data (due to the proclaimed smaller solution space), the production rate of the fermentation products, ethanol, formate and acetate, resulting from the perturbation analysis were compared to the experimental data for the two different models. The result showed that in the mutant model, only 2.8% of the ethanol production values, 50% of the acetate and 7.9% of the formate production values were within a ±10% interval of the measured experimental data. In the case of the wildtype, no flux distribution profile was beyond the ±10% interval of the experimental data. This was expected, as it was described above that there was no observed variation at the pyruvate branching point in the wildtype model.

To find out whether the constraints on the fermentation products are the main contributor to the smaller solution space in the wildtype, the exchange reaction constraints of the four fermentation products of the mutant, namely lactate, formate, ethanol and acetate were integrated into the wildtype model. The model was then again subjected to the perturbation approach, and the result revealed that the sensitivity of the model increased to a level comparable to that of the mutant model. This suggests that the smaller solution space in the wildtype is mainly a result of the specific constraints of these four reactions. Not surprisingly, the four reactions are involved in energy metabolism and the flux ratio of these reactions determines the amount of energy that the system produces.

The result indicates that the functional analysis of the solution space can be very informative when studying biologically relevant phenotypes, as the functional consequences of having large or small solution spaces can be determined.

### 2.5. Analysing the Solution Space Using CoPE-FBA

Using the above described perturbation approach seems to offer a relatively cheap (in computational power) and fast way to investigate the solution space of whole-genome scale models. In the following, we compare its results to another widely used approach—CoPE-FBA. This method is a freely available open source software that guarantees access to the sources. We want to see whether the modules generated by CoPE-FBA can reflect the extent to which each model is constrained and whether they can relate to the ability of the models to reproduce biologically meaningful results.

For this purpose, we analysed the quantitative impact that the constraints have on the size of the solution space by using CoPE-FBA developed by Kelk et al. This software analyses the solution space by defining modules consisting of sets of reactions with variable flux that are linearly independent. The combination of variable fluxes within these modules results in the expansion of the solution space.

We again used the wildtype and mutant model of *E. faecalis,* for which we have the above described range of experimental data (medium composition, metabolite uptake and production fluxes, as well as proteome data). We calculated the solution space of the non-constrained models and compared these to the models constrained with medium composition, metabolite flux data and/or proteome data. Here, a higher number of modules with a lower number of reactions in each would imply a smaller solution space. As can be seen in Table 2, the effect of applying metabolite flux data is surprisingly low, whereas the application of the constraints arising from the proteome data eliminating some of the reactions in the network is—not surprisingly—profound.

Comparing the result of the solution space analysis from CoPE-FBA to the perturbation procedure, it is apparent that although CoPE-FBA successfully captures the quality of reducing the solution space by applying more constraints to the system, it comes short in reflecting the extent of reduction. The perturbation analysis, as discussed above, showed that the integration of metabolic flux data results in a considerable reduction in the sensitivity both in the general term and the functional aspects of the solution space (analysis of the branches). It also revealed that the fully constrained wildtype model has a considerably more limited solution space compared to the fully constrained mutant model. Neither of the two qualities were detected by the CoPE-FBA method. It has to be mentioned that the CoPE-FBA could not be performed fully, as enumerating the number of solutions was computationally infeasible due to the large solution space of the models. We suggest that in addition to being faster, even for large models, the perturbation procedure reflects more aspects of the solution space that can be helpful to also functionally analyse the solution space.

### 2.6. The Influence of ATP Maintenance on the Solution Space

Traditionally, maximal growth is the objective function of choice when computing FBA on microbial genome-scale metabolic models. Under conditions of limiting resources/balanced growth, commonly applied for such studies, maximizing growth intrinsically implies the maximization of growth associated ATP production, and consequently, minimizing non-growth associated ATP consumption. The latter processes are included in genome-scale models in the form of a generic ATPm reaction which refers to all processes that require ATP, but do not yield biomass. Consequently, the reaction boundaries of both reactions can directly contribute to shape the solution space, as the main criteria in FBA is maximizing the growth-associated ATP production (assuming that the objective function is the biomass reaction). As a parameter, ATPm has been shown to be important for the result of FBA and has a pronounced impact on the consistency of biomass production [17].

**Figure 9:**
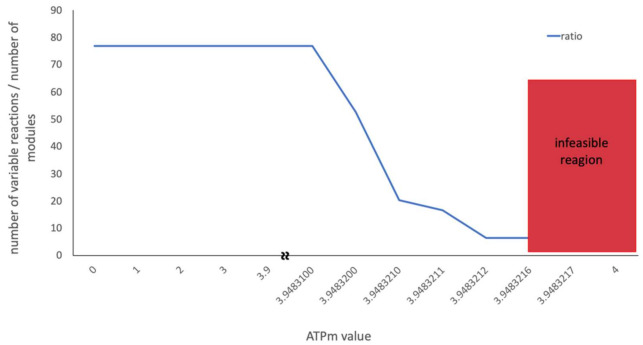
Flux scan of the ATPm value over the feasible region of the wildtype model. The blue line shows the ratio between the number of variable reactions and the number of modules generated by CoPE-FBA.

Due to its central importance, in several studies the ATPm has been experimentally assessed with the aim to constrain the model in a biologically relevant way [19,28]. The inclusion of an experimentally measured ATPm value assures that the minimum required amount of non-growth associated ATP is produced, while growth-associated ATP is maximized. To investigate whether the ATPm value has any direct impact on the solution space, a flux scan was performed on the wildtype model (integrated with metabolic and proteome data) over the whole feasible interval of the ATPm value and the solution space was calculated accordingly using CoPE-FBA (Figure 9).

The result of the scan revealed no significant change in the size of the solution space until the ATPm value was right on the edge of the feasible state. While CoPE-FBA reported a significantly smaller solution space at this point, a closer look at the optimization process revealed that FVA failed to determine the flux interval for several reactions at this point. To make sure this is not a model-specific problem, the same procedure was performed on the mutant model, which resulted in the same kind of artefact. This suggests that when a parameter is tied to the edge of the feasible state (by, e.g., its value being adjusted up to several digits after the decimal during FBA), the optimisation process might fail to calculate the entire feasible interval, resulting in an artefact, which in this case appeared as a smaller solution space.

To test whether this problem relates exclusively to ATP/energetically-involved reactions or not, the procedure was performed on lactate dehydrogenase (as an example of an energetically important reaction which does not contain ATP in its stoichiometry), as well as on ribulose 5-phosphate 3-epimerase (as an example of an energetically nonrelated reaction). The result revealed that the same artefact appeared in the case of both examples, although to different extents (data not shown). Consequently, we suggest that this is a universal characteristic of reactions in a constraint-based model and has to be considered when integrating experimental data into models. This is particularly important for the constraints whose values are obtained from an optimisation process such as ATPm.

The problem of creating a numerical artefact by working at the edge of feasible solutions was discovered in the originally published version of the data integrated *E. faecalis* wt model [7] when using 100% optimality tolerance during FVA. Here, the ATPm value was optimised to be exactly at the edge of feasibility. Subsequently, two ways to avoid the aforementioned problem were investigated. One is to decrease the optimality tolerance of FVA from 100% to a lower value (e.g., 99.9%). This was done in the original publication and results in a higher number of variable reactions and potentially a larger feasible interval for some reactions. Another way to overcome the instabilities on the edge of the feasible state is to change the value of one or a few flux boundaries to a very small extent. So, e.g., when the lower bound of the formate exchange reaction was decreased by 0.05 unit, which also allowed to set back the ATPm value to the originally optimized one, the model was no longer at the edge of feasibility. This strategy is currently used for all of the above described analyses in this study.

Of course, the question is how such an adjustment that avoids the problem of working at the edge of feasibility or having an artificially small solution space influences biologically relevant results in this study or in any other computational study. Therefore, we compared the results of the originally published parametrisation of the model with an optimality tolerance of FVA set to 100% (leading to numerical artefacts as discussed above) with the one where the tolerance is set to 99.9%, as well as with the results of the slightly adjusted model version, also analysed with a tolerance of 100%, as well as 99.9%. Table 3 summarizes the results of FVA of these four settings, revealing a higher number of variable reactions when the tolerance is less strict which is not surprising. In the original model setting, this difference is dramatic. However, as discussed above, this is clearly a numerical artefact due to the inability of FVA to run optimisations in several cases.

We repeated this analysis with our new perturbation approach. The number of sensitive reactions showed a similar, but less dramatic trend (Table 3) (Supplementary Figure S2). Finally, we also analysed the impact of the decreased optimality tolerance of FVA on flux distributions in the biologically relevant cases as described above. Here, no significant difference was observed (Supplementary Figures S3–S5).

### 2.7. Validating the Results Using Models of Other Species

To find out whether the results from the functional analysis of the solution space, as well as the resulting conclusions are specific to the genome-scale metabolic model of *E. faecalis* or rather general to genome-scale metabolic models, the genome-scale models of *S. pyogenes* [20] and *L. lactis* [21] were subjected to some of the analyses performed on *E. faecalis*. For the model of *S. pyogenes*, a previously published genome-scale model together with experimental data [20] was used. Similar to *E. faecalis*, constraints were step-wise integrated into the model, comprising medium composition and metabolite uptake and production rates. Due to the absence of proteome data, two artificial sets of proteome data were generated for *S. pyogenes* (as described in Materials and Methods) to observe the effect of the deactivation of model reactions on the solution space. The two artificial sets of proteome data contain 11 “absent” reactions and are integrated into the model separately. Between the two sets, one set contained the absence of 5 variable reaction and 6 stable reactions (according to the above definition), while the other contained the absence of 10 stable and only 1 variable reaction (based on the FVA of the original model). Here again, the integration of experimental data at each step (medium composition and metabolite uptake and production rates) resulted not only in a lower number of variable but also sensitive reactions (Supplementary Figures S6–S9). The integration of proteome data, i.e., the deactivation of the respective reaction, in both cases resulted again in a decrease in the number of variable (according to FVA) as well as sensitive reactions. However, the integration of the second artificial proteome data set with a higher number of variable reactions had a more pronounced effect compared to the first proteome data set showing a low number of variable reactions.

To further test whether the inactivation of variable reactions (which increases the proportion of stable reactions in the model) has a reproducible effect (decrease) on the number of sensitive reactions, again, artificial proteome data sets, now for the *L. lactis* model were produced so that one set contained 15 stable reactions and the other contained 15 variable ones. While the integration of the first set (stable reactions) decreased the number of sensitive reactions only by 3, the integration of the second set resulted in a decrease of 44 reactions (Supplementary Figure S10). Hence, it can be suggested that while deactivating reactions in a model, such that the proportion of variable reactions decreases (deactivating more variable reactions than stable reactions), the model behaves more robust (an indication of a smaller solution space). Deactivating stable reactions does not essentially have the same effect.

## 3. Discussion

Genome-scale metabolic models are mathematically underdetermined systems with large solution spaces. The solution space comprises all the possible flux distributions that result in the optimum value of the objective function. Flux distribution profiles in any given model are highly variable, mainly due to branches and nonlinearities of the metabolic networks on the one hand, and the lack of sufficient constraints on the other hand. Thus, while satisfying the optimum value of the objective function, two possible flux distributions might be enormously different. To find out about the extent of variation at different levels, genome-scale metabolic models were subjected to perturbations, and the response of the FBA was observed accordingly. In that way, different specific optimal flux distributions became visible and were sampled. It became apparent that there are not only quantitative differences in the respective flux distributions, but also qualitative ones that impact the biological interpretation of the results.

During the analysis, we were, however, also interested in the quantitative differences of sensitivity. We therefore defined a significantly changed flux value to vary more than ±5% compared to the original values. We defined fluxes that only changed to a smaller degree as “robust”. This allowed us in a straightforward manner to analyse the influence of constraints and differences in models w.r.t. the size of the permissive flux intervals. We found that the number of “sensitive” reactions—those that display values beyond ±5% deviation of their original value—is a good indicator of the size of the solution space in genome-scale models. We show that the models with lower numbers of sensitive reactions are able to fit the experimental data better and have (potentially) better predictive characteristics.

The substantial variation in flux distributions obtained by FBA underlines the fact that a reported optimal flux distribution is just one out of many other optimal distributions and therefore the distribution profile that is a result of one run of FBA is arbitrary. It is therefore important that the result of a FBA flux distribution is referred to as ‘an optimal’ instead of ‘the optimal’ flux distribution.

Three further aspects result from our analysis:

1. To decrease the number of biologically inconsistent results, it is vital to integrate biological constraints. In our analysis, generally the integration of proteome data was the most effective in reducing the solution space. However, metabolic flux data on exchange reactions (metabolic uptake and production rates) can also already significantly reduce the solution space (decrease in sensitivity), e.g., at branching points. The fact that the degree of reduction is really case-specific underlines the second point [29].
2. The analysis of the solution space should be taken into account in any study using FBA. As the above cases demonstrate, constraints like metabolite exchange rates, which are arguably one of the more commonly used constraints, can effectively reduce the solution space such that biologically relevant results for certain questions (e.g., specific kind of fermentation) are achieved, but this is not the case for every model/data-set. There are different ways to investigate the solution space. In our study, sampling by perturbation was an easy and informative way to investigate the different optimal flux distributions. We suggest that the functional analysis of the solution space using our perturbation method gives an explicit account for the robustness as well as reliability of genome-scale models. This also enables us to understand which data sets and which biological phenotypes can effectively shrink the solution space and increase the predictability of models.
  However, there are alternative methods for the analysis of the solution space not investigated here, e.g., Monte-Carlo sampling. This approach is mostly used to calculate the probability distribution of individual fluxes as well as to determine correlated reaction sets which can be further used for experimental design [12,14]. While this aims at the probability distribution of individual fluxes, our method is focused on uncovering the uncertainty in the interplay between different metabolic fluxes. As mentioned above, Monte-Carlo sampling enables the calculation of correlated reaction sets, which can be used to select candidate reactions for flux measurements, helping to estimate the flux value of its correlated ones. Nevertheless, our method showed, while correlated, the integration of metabolic fluxes of fermentation products for which the internal reactions were reported to be correlated (e.g., LDH, PFK [12]), does not necessarily result in eliminating the physiologically inconsistent result (see analysis of the pyruvate branching point in the case of the *E. faecalis* mutant). Therefore, the analysis of the solution space using the perturbation procedure helped to yield more information regarding the behaviour of the network as a whole. Another difference between the method presented in this article and Monte-Carlo sampling is a far smaller sample size needed to capture the network response to different metabolic states. While Monte-Carlo sampling needs a large sample size to reveal a comprehensive overview (250,000 data points in [12]), our method uncovers different aspects of the solution space using a far smaller sample size (~10 times the number of variable reactions). However, this also implies that our method does a less complete sampling of the solution space and certain alternative solutions might be overlooked. Moreover, although it is hard to compare the computational performance since our method has a different purpose, we would like to state that our method is fast compared to the Monte-Carlo sampling methods with respect to computational time. The comparison of different Monte-Carlo sampling methods reported that the sampling time spans from 7.64 to 10.67 min, for models of comparable size to our models (especifically the model of *E. faecalis*) using the CHRR method (the most efficient Monte-Carlo method available right now ) on an intel Core i7 at 2.5 GHz as reported by Fallahi and colleagues [15]. In this study, a reduced version of the metabolic models was used, meaning that the reactions carrying no flux were discarded. Therefore, the number of reactions in the case of the four models, iLJ478, iSB619, iHN637 and iJN746 were reduced from 652, 743, 785 and 1054 to 380, 450, 522 and 652, respectively [15]. The perturbation process of our method took between 122 to 175 seconds depending on the model (wildtype or mutant) and how constrained a model was, in the case of the *E. faecalis* model on an Intel Core i5 2.3 GHz, 16 MB memory and HDD hard drive, when the flux distribution profiles were obtained using FBA (on MATLAB). Our method also allows the acquisition of flux distributions using FVA, which takes more time—in this case between 31 to 51 min for the same models on the same hardware setup. Comprehensive information regarding the run time of different models used in the above-mentioned study using CHRR and the perturbation process in this study, as well as the number of metabolites and reactions of each model used can be found in the Supplementary Tables S2a and S2b (Supplementary file 2, sheet: run time comparison). Of course; any additional statistical analysis takes further time.

3. Caution has to be taken if outcomes of FBA are close to the edge of the feasible solution space w.r.t. some parameter, e.g., ATP maintenance. This is at least true when applying methods that are based on FVA, as shown in Section 2.6, since FVA often fails under these conditions and a solution space smaller than the actual space is reported.

Finally, it is important to note that none of the employed methods allows to distinguish very clearly between the absolute solution space of the FBA as such and the practically determined solution space as reported by the numerical methods or software. In our study, there was no qualitative difference between various optimization algorithms with respect to major trends such as the decrease in the number of sensitive reactions following the integration of constraints. However, different numerical solvers/algorithms and/or software surprisingly resulted in minor quantitative differences, e.g., in the number of sensitive reactions, or even major differences, e.g., the average number of reactions reacting to one perturbation. Therefore, in addition to reliable qualitative outcome (the trend of change), the best quantitative indicator for the size of the solution space is the number of sensitive reactions, which only shows slight differences between different algorithms/software.

## 4. Materials and Methods

### 4.1. Models, Experimental Data and Constraints Integration

The genome-scale metabolic models of *E. faecalis* (wildtype) [19] and of a knock-out mutant of glutamine synthase (Δ*glnA*) of *Enterococcus faecalis* [19] were used for the initial analysis. The experimental data was obtained from [7] for the wildtype and [30] for the mutant. The findings reported here were validated using the genome-scale models of *S. pyogenes* [20] and *L. lactis* [21] together with the respective published experimental data. The models with no constraints (denoted by nc throughout the text) had all the upper bounds set to +1000 (representing +infinity) and lower bounds to −1000 (representing −infinity) except for the biomass reaction that was fixed at the intended objective function value. Constraints were integrated at three different steps using the respective experimental data, medium composition, metabolite uptake/secretion rates (organic acids and amino acids) and proteome data. To integrate the medium composition data, the upper and lower boundaries of the respective reactions were adjusted. To integrate the metabolite uptake and secretion rates, the experimentally measured flux value of metabolites was used as the basis and a tolerance value of 40% was considered to account for measurement error. Consequently, 20% were added to the measured value and used as the upper bound and the subtraction of 20% was used as the lower bound [7]. To integrate proteome data, the flux value of the reactions whose respective proteins were not detected by the proteomics experiment were set to zero [7]. The complete list of constraints used for each model, together with the list of metabolites and genes can be found in the supplementary file 2. In summary, the *E. faecalis* model contains 709 reactions (in the case of the wildtype, 708 in the case of the mutant), 644 metabolites and 688 genes. The model of *S. pyogenes* contains 576 reactions, 558 metabolites and 481 genes. The model of *L. lactis* contains 754 reactions, 650 metabolites and 516 genes.

### 4.2. Perturbation Procedure

**Figure 10:**
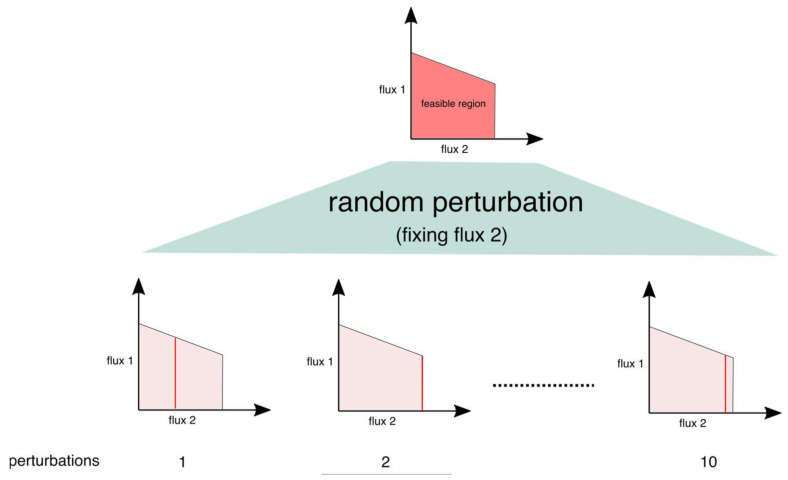
Perturbation procedure to determine the robustness of FBA/FVA outcome. The figure shows how fixing one reaction at various random values results in a different range for flux combinations between two fluxes.

The perturbation procedure was used to determine the effect of alternative optimal values taken by each reaction on the flux distribution profile of the network. Given the fact that parameters within the permissible flux interval calculated by FVA can result in the optimal value of the objective function, we wanted to find out how each point in the permissible interval affects the quantitative and qualitative flux distribution in the network. The steady-state flux distribution of a constraint-based model can be obtained by:

(1) S⋅v = 0,

where *S* represents the stoichiometric matrix and *v* is the vector of flux distribution. Here, the maximum and minimum values of individual reactions can be obtained using FVA [10] by maximizing the objective function and using the respective value as an additional constraint:

(2) $$Maxf^{T}v,\;s.t.S\cdot v\;=\;0,\;v_{min}\;\leq \;v\;\leq \;v_{max}$$

where *f* is the objective function vector and *v_max_* and *v_min_* are the vectors of maximum and minimum allowable flux values, respectively, for each reaction. Using this characteristic of constraint-based models, the perturbation procedure we proposed is based on the idea that a change in the flux value of a reaction would result in a different combination of fluxes in the network, as shown in the Figure 10, in the example of two flux combinations:

Consequently, the robustness of model predictions, whether they are qualitative biological phenotypes, or flux values/range for a several reactions can be examined and the overall predictability of the model can be determined.

All analyses were done using the FBA perturbation toolbox which is available on Github [31], developed for this project. The toolbox was primarily developed for MATLAB, but a Python version is available as well. All analyses in this paper were done using the Cobra toolbox version 3.1 [32,33] as the platform for constraint-based modelling on MATLAB R2018a [34] on MacOS Mojave version 10.14.6 (Apple.com (accessed on 14 February 2020)). To perform the perturbations, the biomass reaction was set as constraint and fixed at the intended value (lower and upper bounds having the same value). Afterwards, the flux boundaries of variable reactions were determined by FVA (using CPLEX 12.8.0 [35] (ibm.com (accessed on 27 February 2020)) as the solver) with an optimality percentage of 100% or 99% as indicated in the text. The permissible interval size for the flux of each reaction as calculated by FVA was determined and a threshold of 10^−6^ was applied to consider a reaction as variable. Next, for each variable reaction, 10 random values within the determined permissible interval were selected using the ‘rand’ function in MATLAB. The rand function yields a single uniformly distributed number within the given interval. The respective reaction was fixed at the given random value (lower and upper bounds having the same value) and the flux distribution profile was recalculated each time using FVA. For validation purposes, the analysis was repeated and the flux distribution was obtained with FBA (using CPLEX and GLPK 4.65 [36]) in COBRA. The analysis was also repeated using PySCeS-CBMPy 0.8.0 [22] on Python 2.7 [37] and the flux distribution profiles were again obtained using FVA (using CPLEX as solver). The obtained flux distribution for each reaction was then compared to the original flux distribution and a flux value was considered to change significantly, if it was altered beyond ±5% of the original flux value. In the cases where the original flux value was zero, the threshold was set to 10^−6^.

### 4.3. Analysing the Solution Space Using CoPE-FBA

A standard analysis of the solution space was performed using CoPE-FBA with CPLEX as solver [11]. For this purpose, FVA was performed and variable reactions were determined with the interval size of 10^−6^ as a threshold. Afterwards, flux modules, which are the sets of variable reactions that are linearly independent, were determined. The modules were then used to analyse the solution space.

## Figures and Tables

**Table 1:** Number of variable reactions in differently constrained genome-scale models of *E. faecalis* wildtype (wt) and Δ*glnA* mutant (mt). Here, “nc” indicates model version without any constraints, “med” indicates integration of medium composition, “met” the additional integration of data on metabolite uptake and release and “pro” the additional integration of proteome data.

Model Name	Number of Variable Reactions Variability > 10^−6^	Number of Variable Reactions Variability > 10^−3^	No of Reactions
mt + nc	397	397	708
mt + med	362	340	708
mt + med + met	347	315	708
mt + med + met + pro	298	289	708
wt + nc	398	398	709
wt + med	363	341	709
wt + med + met	362	340	709
wt + med + met + pro	307	85	709

**Table 2:** The number of reactions in the existing modules in each model when their solution space was analysed with CoPE-FBA.

Model Name	Number of Reactions in Each Module
mt + nc	399
mt + med	360, 4
mt + med + met	345, 4
mt + med + met + pro	286, 5, 4, 4
wt + nc	400
wt + med	361, 4
wt + med + met	360, 4
wt + med + met + pro	295, 5, 4, 4

**Table 3:** Number of variable reactions according to FVA (with the interval size larger than 10^−6^), number of sensitive reactions according to the solution space inspection procedure (perturbation analysis), and the number of reactions in existing modules in each model (CoPE-FBA). All three methods were used with two optimality tolerance values (100% and 99.9%) and the respective results were compared.

	Wt + Med + Met + Pro-Edge		Wt + Med + Met + Pro	
optimality tolerance	100	99.9	100	99.9
FVA	209	387	307	387
#reactions, sensitive to perturbation	94	137	137	151
#modules according to CoPE-FBA	4, 13, 7, 5, 4, 4, 12, 3	295, 5, 4, 4	295, 5, 4, 4	295, 5, 4, 4

## Data Availability

Data is contained within the Supplementary Materials.

## Supplementary Materials

The following supporting information can be downloaded at: https://www.mdpi.com/article/10.3390/metabo12010043/s1, Figure S1: Comparison between the sensitivity frequency and the FVA interval size, Figure S2: The proportion of sensitive reactions with respect to perturbations in other reactions in the genome-scale models of *E. faecalis* wildtype (wt) and Δ*glna* mutant (mt), when the perturbation procedure was performed with opt-percentage of 99.9 in FVA, Figure S3: The relative flux distribution at the carbohydrate branching point in the two genome-scale models of *E. faecalis* with an opt-percentage of 99.9 in FVA, Figure S4: The relative flux distribution at the branching point in serine metabolism in the two studied genome-scale models of *E. faecalis* with an opt-percentage of 99.9 in FVA, Figure S5: The relative flux distribution at the branching point in glutamine metabolism in the two studied genome-scale models of *E. faecalis* with opt-percentage of 99.9 in FVA, Figure S6: The proportion of sensitive reactions with respect to perturbations in other reactions in the genome-scale models of *S. pyogenes*, Figure S7: The relative flux distribution through the carbohydrate branchpoint in the genome-scale models of *S. pyogenes*, Figure S8: The relative flux distribution through the serine metabolism in the genome-scale model of *S. pyogenes*, Figure S9: The relative flux distribution through a branch point in glutamine metabolism in the genome-scale model of *S. pyogenes*, Figure S10: The proportion of sensitive reactions with respect to perturbations in other reactions in the genome-scale models of *L. lactis* wildtype, Supplementary file 1, Table S1: Statistical analysis of the results from the perturbation process, Supplementary file 2: The complete list of reactions, metabolites, GPR of each model; plus the list of constraints used for each model, Supplementary file 2, Table S2a: The run time of different models using CHRR algorithm, Supplementary file 2, Table S2a: The run time of different models using the perturbation process for FBA and FVA.
