# Supplementary material for: Inspecting the Solution Space of Genome-Scale Metabolic Models
Source: Metabolites. 2022 Jan 5;12(1):43. doi: 10.3390/metabo12010043 (PMC8779308; doi:10.3390/metabo12010043)
Supplement: Supplementary file 1 [file metabolites-12-00043-s001.zip › Supplementary-figures.pdf]

Supplementary figures

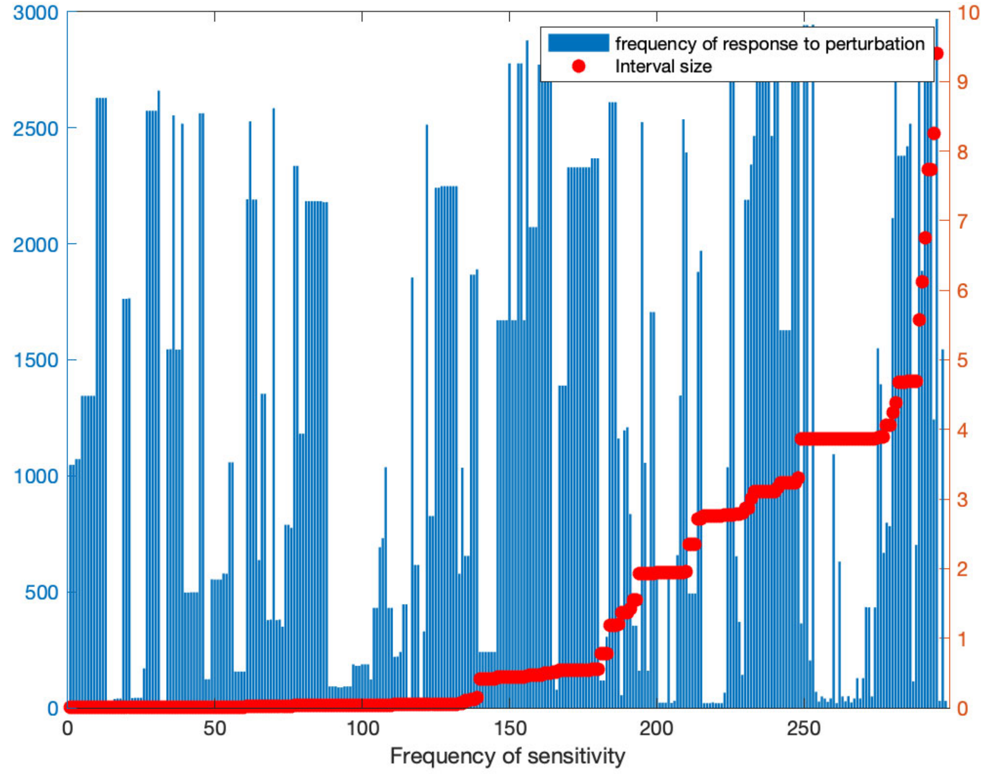

Figure S1. Comparison between the frequency of response to perturbation (sensitivity frequency) and the FVA interval size in the mutant model integrated with medium composition, metabolic and proteome data. The graph shows that the frequency of sensitivity is not correlated with the FVA interval size.

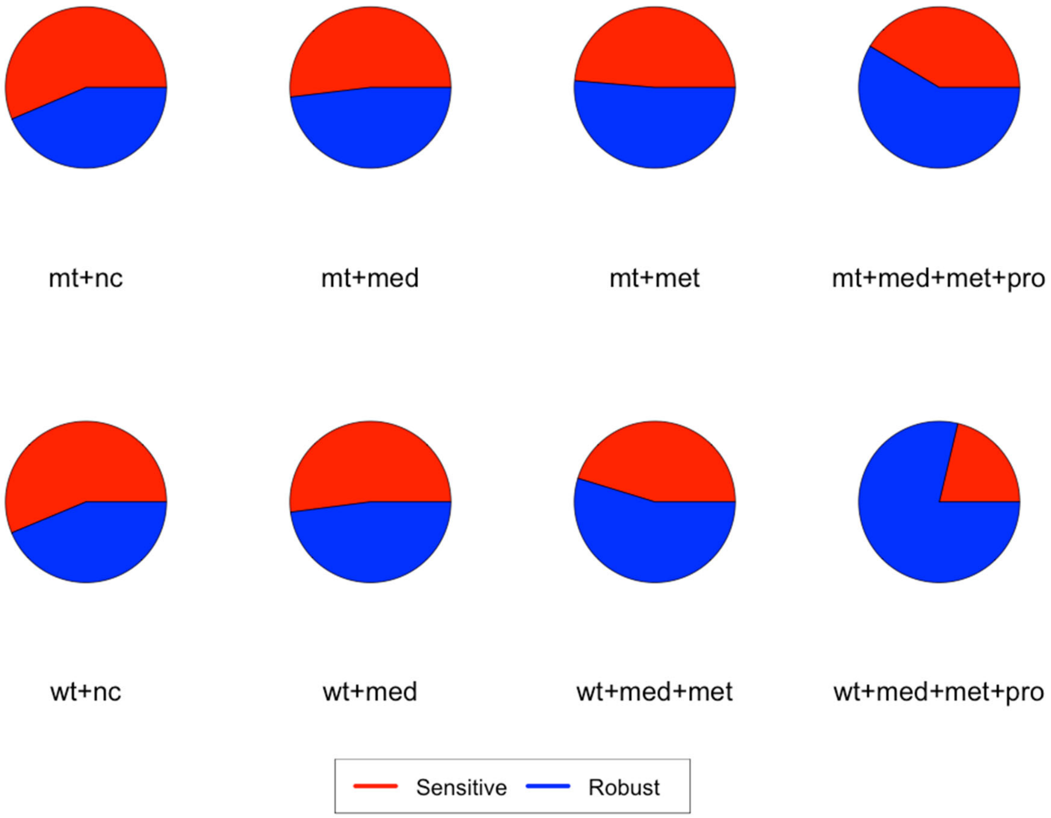

Figure S2. The proportion of sensitive reactions with respect to perturbations in other reactions in the genome-scale models of E. faecalis wildtype (wt) and Δglna mutant (mt), when the perturbation procedure was performed with opt-percentage of 99.9 in FVA. The integration of constraints (none, medium composition, metabolic and proteome data, from left to right) into the model results in reducing the number of sensitive reactions (red) and increasing the number of robust reactions (blue).

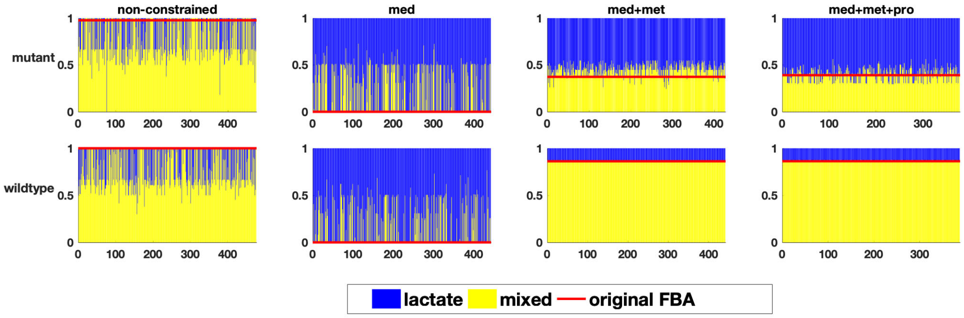

Figure S3. The relative flux distribution at the carbohydrate branching point, resulting in homolactic or mixed acid fermentation in the two genome-scale models of E. faecalis with an opt-percentage of 99.9 in FVA.

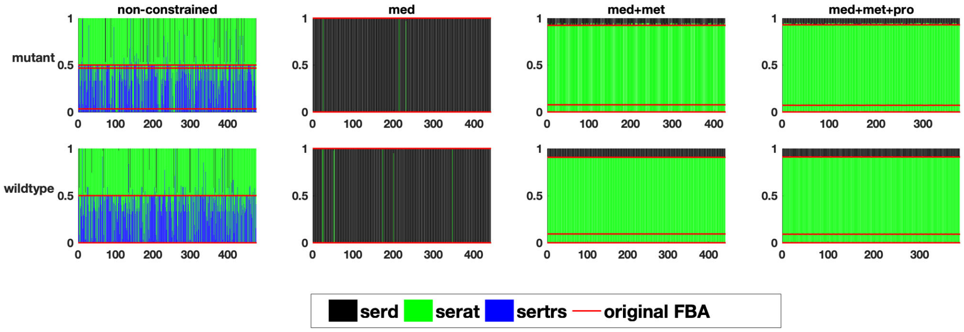

Figure S4. The relative flux distribution at the branching point in serine metabolism in the two studied genome-scale models of E. faecalis with an opt-percentage of 99.9 in FVA, resulting in the production of acetyl serine, seryl-tRNA or serine secretion.

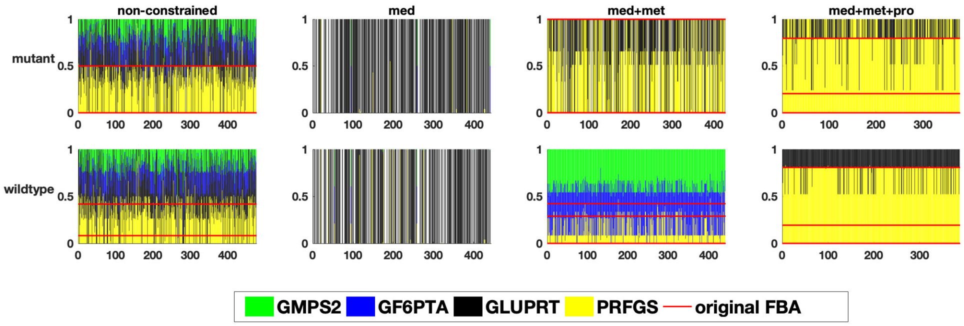

Figure S5. The relative flux distribution at the branching point in glutamine metabolism in the two studied genome-scale models of E. faecalis with opt-percentage of 99.9 in FVA, resulting in the distribution of glutamine in different pathways, namely amino acid, purine and pyrimidine metabolism.

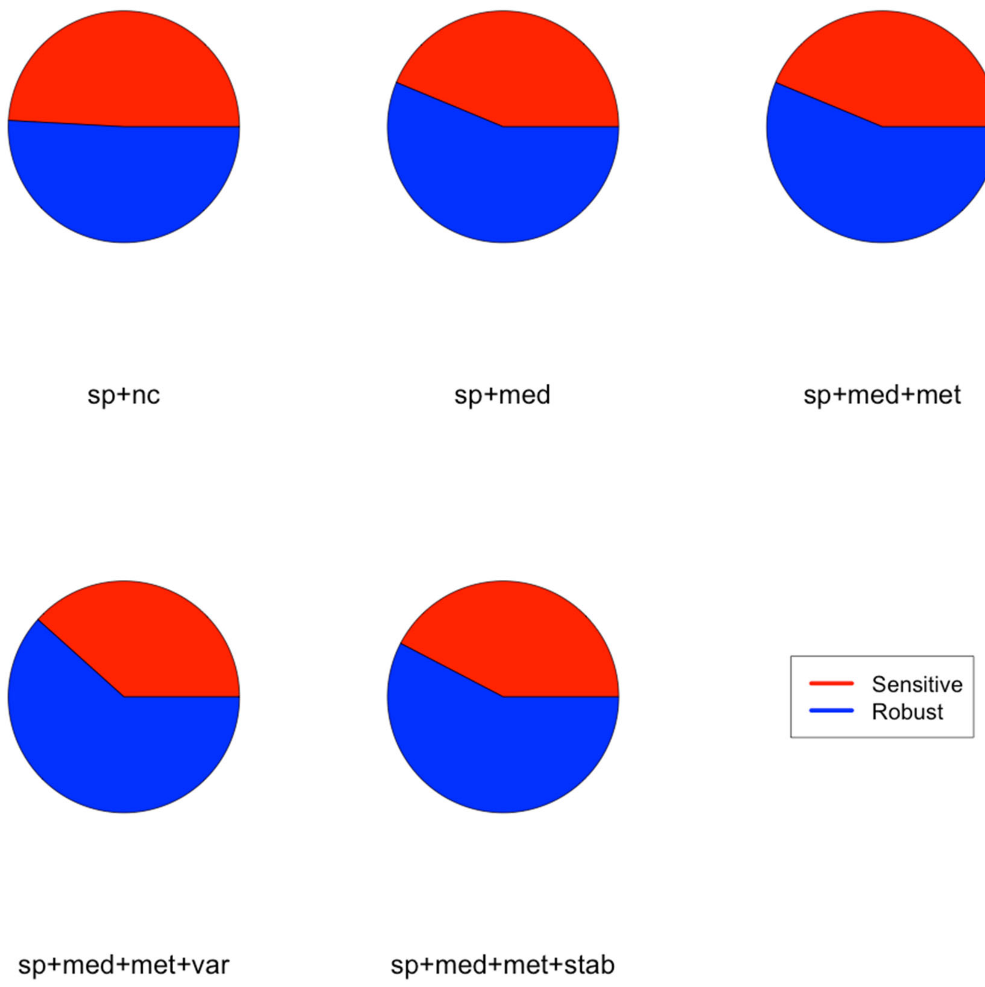

Figure S6. The proportion of sensitive reactions with respect to perturbations in other reactions in the genome-scale models of S. pyogenes. In the two charts on the bottom, "var" refers to the case when more variable reactions were deactivated than stable reactions, and "stab" refers to the case when more stable reactions were deactivated than variable reactions. The integration of constraints (none, medium composition, metabolic and proteome data, from left to right) into the model results in reducing the number of sensitive reactions (red) and increasing the number of robust reactions (blue). The deactivation of a

higher number of variable reactions had a slightly more impact on the decrease of the number of sensitive reactions.

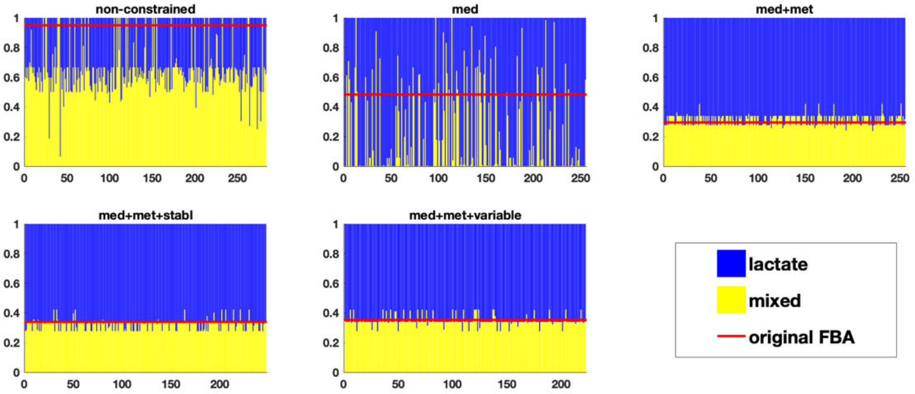

Figure S7. The relative flux distribution through the carbohydrate branchpoint, resulting in homolactic or mixed acid fermentation in the genome-scale models of S. pyogenes.

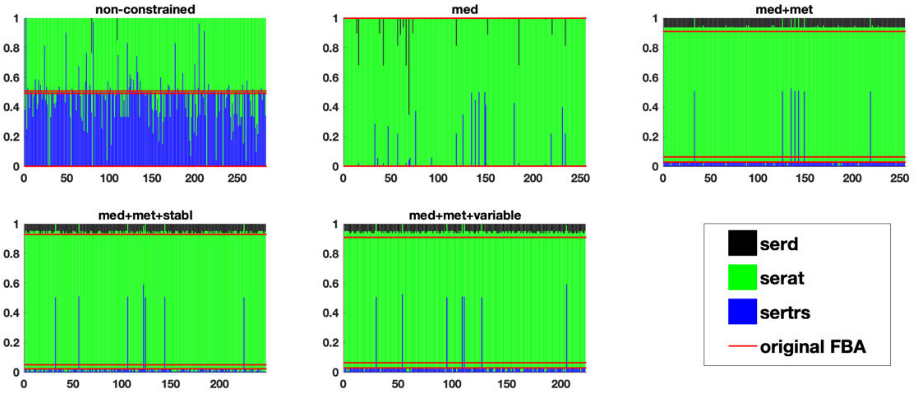

Figure S8. The relative flux distribution through the serine metabolism in the genome-scale model of S. pyogenes, resulting in the production of acetyl serine, or seryl-tRNA or serine secretion.

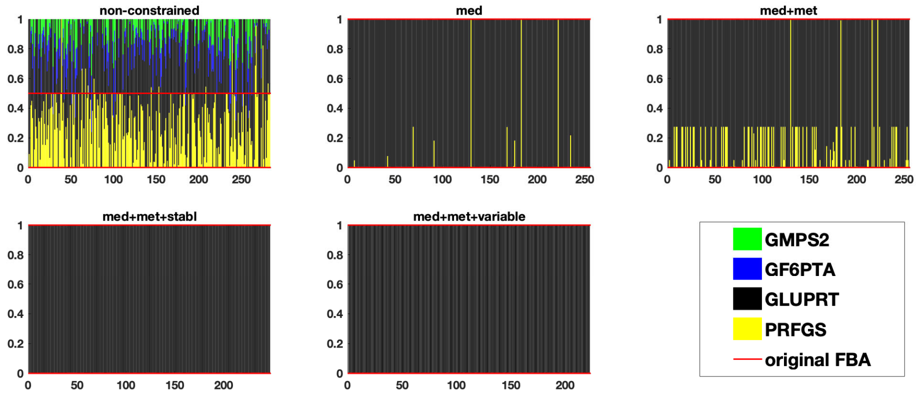

Figure S9. The relative flux distribution through a branch point in glutamine metabolism in the genome-scale model of S. pyogenes, resulting in the distribution of glutamine in different pathways, namely amino acid, purine and pyrimidine metabolism.

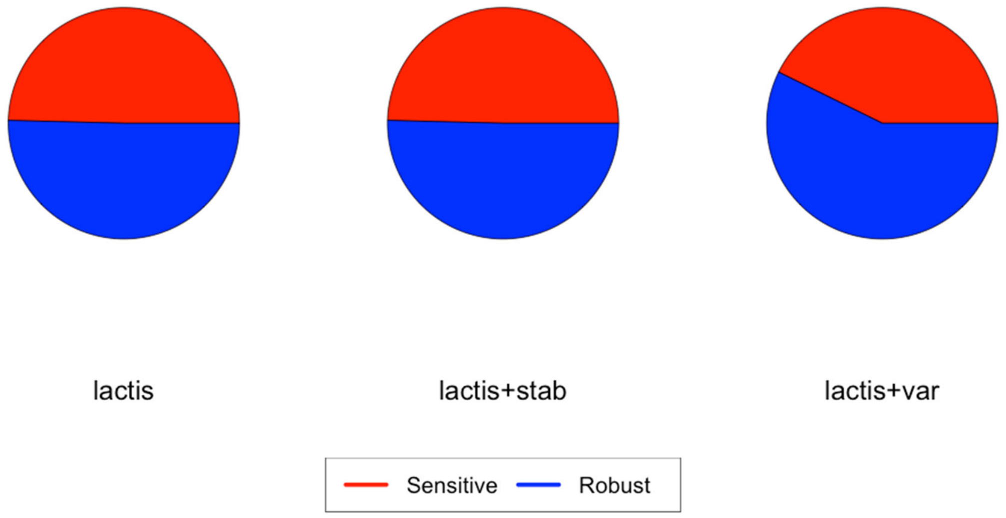

Figure S10. The proportion of sensitive reactions with respect to perturbations in other reactions in the genome-scale models of L. lactis wildtype. “stab” refers to the case when only stable reactions were deactivated, while “var” refers to the case when only variable reactions were deactivated. Not surprisingly, the deactivation of variable reactions had more impact on the number of sensitive reactions.
